# Inflammatory and Coagulation Marker Changes in PMX-DHP-Treated COVID-19 Patients

**DOI:** 10.7759/cureus.78836

**Published:** 2025-02-10

**Authors:** Akinari Tsukada, Daisuke Katagiri, Shinyu Izumi, Junko Terada-Hirashima, Yosuke Shimizu, Yukari Uemura, Masaaki Toda, Taro Yasuma, Corina N D'Alessandro Gabazza, Hajime Fujimoto, Tetsu Kobayashi, Esteban Cesar Gabazza, Masaya Sugiyama, Eisei Noiri, Shinji Abe, Arata Azuma, Haruhito Sugiyama

**Affiliations:** 1 Respiratory Medicine, National Center for Global Health and Medicine, Tokyo, JPN; 2 Nephrology, National Center for Global Health and Medicine, Tokyo, JPN; 3 Center for Clinical Sciences, National Center for Global Health and Medicine, Tokyo, JPN; 4 Immunology, Mie University Faculty and Graduate School of Medicine, Tsu, JPN; 5 Pulmonary and Critical Care Medicine, Mie University Faculty and Graduate School of Medicine, Tsu, JPN; 6 Viral Pathogenesis and Controls, National Center for Global Health and Medicine, Tokyo, JPN; 7 National Center Biobank Network, National Center for Global Health and Medicine, Tokyo, JPN; 8 Respiratory Medicine, Tokyo Medical University Hospital, Tokyo, JPN; 9 Respiratory Medicine, Nippon Medical School, Musashi Kosugi Hospital, Kawasaki, JPN

**Keywords:** coagulation, corisin, coronavirus disease 2019 (covid-19), inflammatory cytokines, polymyxin b hemoperfusion

## Abstract

Introduction

Direct hemoperfusion using polymyxin B-immobilized polystyrene fiber column (PMX-DHP) removes endotoxin and inflammatory mediators from the blood. This study aimed to evaluate the changes in the levels of cytokines, coagulation factors, and a microbiota-derived proapoptotic peptide in COVID-19 patients treated with PMX-DHP.

Methods

We conducted a multicenter, prospective, single-arm interventional study of 21 oxygen-requiring patients with COVID-19 admitted between September 28, 2020, and March 31, 2022. PaO_2_/FiO_2_ (P/F) ratio and biomarkers of inflammation, fibrosis, coagulation, and microbiota-derived peptide were analyzed on PMX-DHP treatment days 1, 4, and 15.

Results

The P/F ratio significantly improved on Day 4 (87.1, 95% CI: 14.8-159.3) and Day 15 (140.6, 95% CI: 56.2-224.9) compared to baseline values. Among the inflammatory cytokines, IL-8 and IL-10 levels significantly decreased on Day 15 (−8.5, 95% CI: −13.4 to −3.5) and Day 4 (−2.3, 95% CI: −5.2 to 0.5) respectively compared to baseline values. Regarding coagulation markers, levels of thrombomodulin increased on Day 4 (1.1, 95% CI: 0.4-1.7) and Day 15 (0.8, 95% CI: 0.3-1.4), and those of tissue plasminogen activator-plasminogen activator-1 significantly decreased on Day 15 (−35.1, 95% CI: −57.6 to −12.6). Microbiota-derived corisin levels significantly decreased on Day 4 (−1740.6, 95% CI: −2860.2 to −621.0) and Day 15 (−1436.7, 95% CI: −2615.8 to −257.6).

Conclusion

Our study revealed improvement in the P/F ratio and the time course of various biomarkers in COVID-19 patients treated with PMX-DHP.

## Introduction

Coronavirus disease 2019 (COVID-19) rapidly became a global pandemic after the first patient was reported in 2019, and the development of various treatments and vaccines has contributed to decreasing trends in the severity and mortality rates. However, as of July 2023, more than 6 million deaths have been reported worldwide [1]. Many studies have shown that COVID-19 patients with progressive disease frequently develop acute respiratory distress syndrome (ARDS), which is triggered by a cytokine storm, and this is a major cause of mortality [2,3]. Compared to healthy individuals, COVID-19 patients have higher levels of inflammatory cytokines, such as interleukin-6 (IL-6), IL-10, and tumor necrosis factor-α (TNF-α) [4,5]. Furthermore, due to diffuse alveolar damage, COVID-19 is associated with a high incidence of pulmonary thrombosis [6], characterized by the impairment of alveolar epithelial and capillary endothelial cells, which leads to the formation of ground-glass opacities and microthrombi that increases the disease severity.

Direct hemoperfusion using polymyxin B-immobilized polystyrene fiber column (PMX-DHP) is a therapeutic approach for removing endotoxins produced by gram-negative bacilli during septic shock [7]. PMX-DHP has been reported to have a positive effect in various conditions, including acute exacerbation of idiopathic pulmonary fibrosis (IPF) and diseases associated with diffuse alveolar damage, and acts by improving oxygenation and prognosis [8,9]. Besides endotoxin removal, PMX-DHP can decrease the hematological expression of inflammatory mediators, such as IL-6 and high-mobility group box 1 (HMGB-1) [10,11]. Based on these beneficial effects, the use of PMX-DHP in COVID-19 patients has been reported in several studies, which suggested that PMX-DHP can improve oxygenation and clinical outcomes [12,13].

Nonetheless, temporal changes and variations in the levels of cytokines, chemokines, coagulation factors, and other factors in response to PMX-DHP treatment for COVID-19 patients remain largely unelucidated. We conducted a multicenter, prospective, single-arm interventional study to evaluate the efficacy and safety of PMX-DHP in COVID-19 patients requiring oxygen therapy and reported that while the improvement rate on day 15 did not differ between PMX-treated patients and historical controls, the deterioration rate was 0.38 times lower in the PMX-treated group [14].

In this study, we aimed to perform an exploratory analysis in COVID-19 patients requiring oxygen therapy to investigate the temporal changes in inflammatory cytokines, coagulation activation markers, and the microbiota-derived peptide corisin after PMX-DHP treatment.

## Materials and methods

Study design

We conducted a multicenter, prospective, single-arm interventional study to assess the efficacy and safety of PMX-DHP in COVID-19 patients [14]. Patients who tested positive for COVID-19 in real-time reverse transcription-polymerase chain reaction (PCR) testing of nasal swabs and were admitted to the National Center for Global Health and Medicine, Tokyo, Japan and Tokyo Medical University Hospital, Tokyo, Japan between September 28, 2020, and March 31, 2022 were enrolled.

In this study, we measured changes in the ratio of partial pressure of oxygen in arterial blood to the fraction of inspiratory oxygen concentration (P/F) ratio and various biomarkers related to inflammation and fibrosis (IL-6, IL-8, IL-10, periostin, periostin monomer, TNF-α, HMGB-1, and thymus- and activation-regulated chemokine [CCL17]), coagulation activation markers (D-dimer, thrombin-antithrombin complex, soluble fibrin monomer, thrombomodulin, tissue-plasminogen activator/plasminogen activator inhibitor-1 complex), and the microbiota-derived proapoptotic peptide corisin from baseline to the period after PMX-DHP treatment initiation.

Study population

The inclusion criteria were as follows: 1) confirmed diagnosis of severe acute respiratory syndrome coronavirus (SARS-CoV-2) infection by PCR or loop-mediated isothermal amplification (LAMP) within the past week, 2) presence of pneumonia findings on chest imaging, 3) P/F ratio of 300 or less or an SpO2 of 93% (room air) or less, 4) requirement of oxygen supplementation or any form of respiratory support, including nasal high-flow oxygen therapy, noninvasive mechanical ventilation, or invasive mechanical ventilation, 5) presence of respiratory distress that cannot be explained by other conditions (heart failure or renal failure), 6) aged 16 years or more at the time of obtaining consent, and 7) written consent obtained from the individual or their legal guardian. Patients meeting inclusion criteria were consecutively enrolled to minimize selection bias.

The exclusion criteria were as follows: 1) severe and progressive multiorgan failure (as determined by the physician in charge), 2) P/F ratio of 100 or less, 3) extracorporeal membrane oxygenation, 4) hospitalization for more than 15 days, 5) platelet count below 20,000/µL, 6) recent treatment with cytotoxic or biological therapy (anti-interleukin-1 [IL-1], anti-IL-6 [tocilizumab or sarilumab], T-cell or B-cell targeted therapy [rituximab, others], tyrosine kinase inhibitors, or interferon) within four weeks prior to consent, 7) therapy with TNF inhibitors within two weeks prior to consent, 8) therapy with convalescent plasma or intravenous immunoglobulin (IVIg) for COVID-19, and 9) individuals who were deemed unsuitable for the study by the principal investigator or co-investigators.

PMX-DHP intervention

A temporary blood access catheter was inserted, and extracorporeal circulation was established. PMX-DHP was performed using Toraymyxin PMX-20R (Toray, Tokyo, Japan) at a blood flow rate of 60-120 mL/min for 3-6 h for 2-3 days for each patient. Nafamostat mesylate (30-40 mg/h) or 40-50 U/kg/h low-molecular-weight heparin (LMWH) was used as anticoagulation therapy. In the event of circuit coagulation, a new circuit was primed and re-established after the blood was returned. Heparin and nafamostat mesylate were used for anticoagulation therapy as part of the hemodialysis and PMX therapy, respectively, as indicated in the package insert. Treatment with heparin and nafamostat mesylate was not considered part of the study intervention. The blood flow rate and duration were determined based on patient weight, hemodynamic stability, and attending physician discretion.

Concomitant medications

No restrictions have been imposed on antiviral therapy for COVID-19. In general, no alterations were made to the antiviral therapy during the study period. Cytotoxic or biological therapy (anti-interleukin-1 [IL-1], anti-IL-6 [sarilumab], T- or B-cell-targeted therapy [e.g., rituximab], tyrosine kinase inhibitors, or interferon), TNF inhibitors, convalescent plasma, or IVIg for COVID-19 were not allowed. The concomitant use of steroids, tocilizumab, and baricitinib was permitted.

Corticosteroid treatment

The attending physician determined the dosage of corticosteroids and options such as steroid pulse therapy (methylprednisolone 500-1000 mg/day for three days), continuous infusion of hydrocortisone at a rate of 10 mg/h for seven days, and daily administration of 6 mg dexamethasone for 10 days were chosen. Each attending physician evaluated the corticosteroid-tapering regimen.

Biochemical analysis

**Figure 1:**
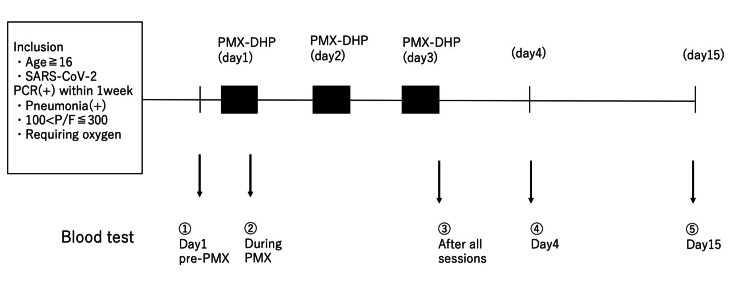
Flowchart depicting the treatment and blood tests In this study, a total of 21 patients received PMX-DHP therapy.

Figure 1 depicts the flow of the PMX-DHP intervention and blood examinations. The levels of thrombin-antithrombin complex (TAT), soluble fibrin (SF), thrombomodulin, and tissue plasminogen activator-plasminogen activator-1 (t-PA-PAI-1) complex were measured by BML Incorporation (Tokyo, Japan). Periostin and HMGB-1 levels were measured by Shino-Test Corporation (Tokyo, Japan). Corisin levels were measured using an enzyme immunoassay as described previously [15].

Laboratory personnel conducting biomarker assays were blinded to treatment time points to reduce potential measurement bias. As a standard procedure, blood samples were collected in the morning under fasting conditions to minimize variability in cytokine and coagulation marker levels. A multiplex cytokine assay was performed to determine the cytokine and chemokine levels using a magnetic 17-plex multiplex assay (Bio-Plex Pro human cytokines GI 17-plex kit, Bio-Rad Laboratories, Inc., Hercules, CA, USA), according to the manufacturer’s instructions. Briefly, serial pre- and post-treatment samples were assayed using a BioRad 3D and Bio-Plex Pro Wash Station equipped with a magnetic manifold. Cytokine levels were derived from the standard run for each assay plate and reported as the serum cytokine levels (in pg/mL). The cytokines and chemokines tested included granulocyte colony-stimulating factor (G-CSF), granulocyte-macrophage colony-stimulating factor (GM-CSF), interferon-γ (IFN-γ), IL-1 beta (IL-1β), IL-2, IL-4, IL-5, IL-6, IL-7, IL-8, IL-10, IL-12 (p70), IL-13, IL-17, monocyte chemoattractant protein 1 (MCP-1), macrophage inflammatory protein (MIP)-1 beta (MIP-1β), and TNF-α. In addition to the 17-plex assay, CCL17 and IFN-lambda 3 (IFN-λ3) levels were measured using the HISCL-5000 analyzer (Sysmex Corporation, Kobe, Japan). In the analysis of biomarkers, values that were out of range were treated as missing. With regard to outliers, considering the small sample size, all biomarker data were included in the analysis after being verified by clinicians to ensure they fell within biologically plausible ranges.

Statistical analysis

Continuous variables were expressed as the mean ± standard deviation (SD) or the median and range as appropriate. Categorical variables were expressed as counts and percentages. Temporal changes in some variables were displayed as box-and-whisker plots. Differences in mean values between different time points and the corresponding 95% confidence intervals [CIs] were presented. A difference was considered statistically significant if the 95% CI did not include zero. For missing data, we did not perform any imputations and used the observed data for our analysis. Outliers were identified using interquartile range (IQR) criteria and excluded from analysis if deemed biologically implausible. All statistical analyses were performed using R version 4.3.1 (R Foundation for Statistical Computing, Vienna, Austria).

## Results

Background characteristics of patients

The patients’ background characteristics are shown in Table 1. Most patients were male, and the median age in this cohort was 55 years. The median duration since symptom onset was approximately 10 days. This clinical study was conducted when COVID-19 vaccination was not widely available. Thus, 18 of the patients (85.7%) were unvaccinated. Baseline endotoxin levels were below the normal range in most patients. Two patients (9.5%) were intubated at enrollment, and seven patients (33.3%) required nasal high-flow oxygen therapy or noninvasive mechanical ventilation. Table 2 displays the medications that the patients received during their hospital stay. Remdesivir was administered to 18 of the patients (85.7%), and all patients received corticosteroids.

**Table 1:** Baseline characteristics of the study cohort The data shows the patient's gender, age, BMI, oxygen status (SpO2, FiO2), and blood test results (D-dimer, CRP, LDH, Endotoxin) at the time of enrollment. "Days from onset" refers to the number of days from the appearance of COVID-related symptoms (e.g., fever) to the time of enrollment. "Vaccination history" indicates whether the patients had received a COVID-19 vaccine. Regarding "Status at baseline," the numbers of patients receiving nasal high-flow oxygen therapy or non-invasive positive pressure ventilation are reported collectively, along with the number of patients on invasive mechanical ventilation. The patients receiving oxygen supplementation via cannulas or masks are categorized under "requiring supplemental oxygen". SD: standard deviation, BMI: body mass index, SpO2: oxygen saturation of peripheral artery, FiO2: fraction of inspiratory oxygen, CRP: C-reactive protein, LDH: lactate dehydrogenase

Variables		PMX-DHP patients (N=21)
Sex	Male	18 (85.7)
	Female	3 (14.3)
Age, years	n	21
	mean (SD)	58.1 (11.2)
	median (range)	55.0 (40–79)
BMI	n	15
	mean (SD)	25.4 (3.4)
	median (range)	25.0 (19.8–31.5)
Days from onset	n	21
	mean (SD)	9.9 (3.0)
	median (range)	10.0 (3.0–15.0)
Vaccination history	Vaccinated	3 (14.3)
	Unvaccinated	18 (85.7)
SpO_2_ (%)	n	21
	mean (SD)	90.9 (3.2)
	median (range)	91.0 (80–97)
FiO_2_ (%)	n	21
	mean (SD)	47.3 (15.9)
	median (range)	44.0 (24.0–71.7)
D-dimer (μg/mL)	n	21
	mean (SD)	4.2 (7.9)
	median (range)	1.4 (0.0–33.5)
CRP (mg/dL)	n	21
	mean (SD)	7.8 (7.1)
	median (range)	5.7 (1.6–34.4)
LDH (U/L)	n	21
	mean (SD)	446.4 (185.7)
	median (range)	372.0 (260–858)
Endotoxin (pg/mL)	n	20
	≦1.0	18 (85.7)
	1.6	1 (4.8)
	3.5	1 (4.8)
Status at baseline	Hospitalized, requiring supplemental oxygen	12 (57.1)
	Hospitalized, requiring nasal high-flow oxygen therapy, noninvasive mechanical ventilation, or both	7 (33.3)
	Requiring invasive mechanical ventilation	2 (9.5)

**Table 2:** Concomitant drugs used during hospitalization The table presents the usage status of concomitant medications, including remdesivir, tocilizumab, baricitinib, heparin, and corticosteroids. For corticosteroids, the maximum dosage converted to methylprednisolone equivalents is shown, along with the number of patients who received dexamethasone.

Drugs	Usage	PMX-DHP patients (N=21)
Remdesivir	Not used	3 (14.3)
	Used	18 (85.7)
Tocilizumab	Not used	18 (85.7)
	Used	3 (14.3)
Baricitinib	Not used	13 (61.9)
	Used	8 (38.1)
Heparin	Not used	3 (14.3)
	Used	18 (85.7)
Corticosteroid	Not used	0 (0.0)
	Used	21 (100.0)
Maximum dose of steroid	250 mg/day ≤Methylprednisolone ≤1000 mg/day (or equivalent dose)	13 (61.9)
	60 mg/day ≤Methylprednisolone <250 mg/day (or equivalent dose)	2 (9.5)
	Methylprednisolone <60 mg/day (or equivalent dose)	1 (4.8)
	Dexamethasone 6.0–6.6 mg/day	5 (23.8)

P/F ratio and inflammatory markers

**Figure 2:**
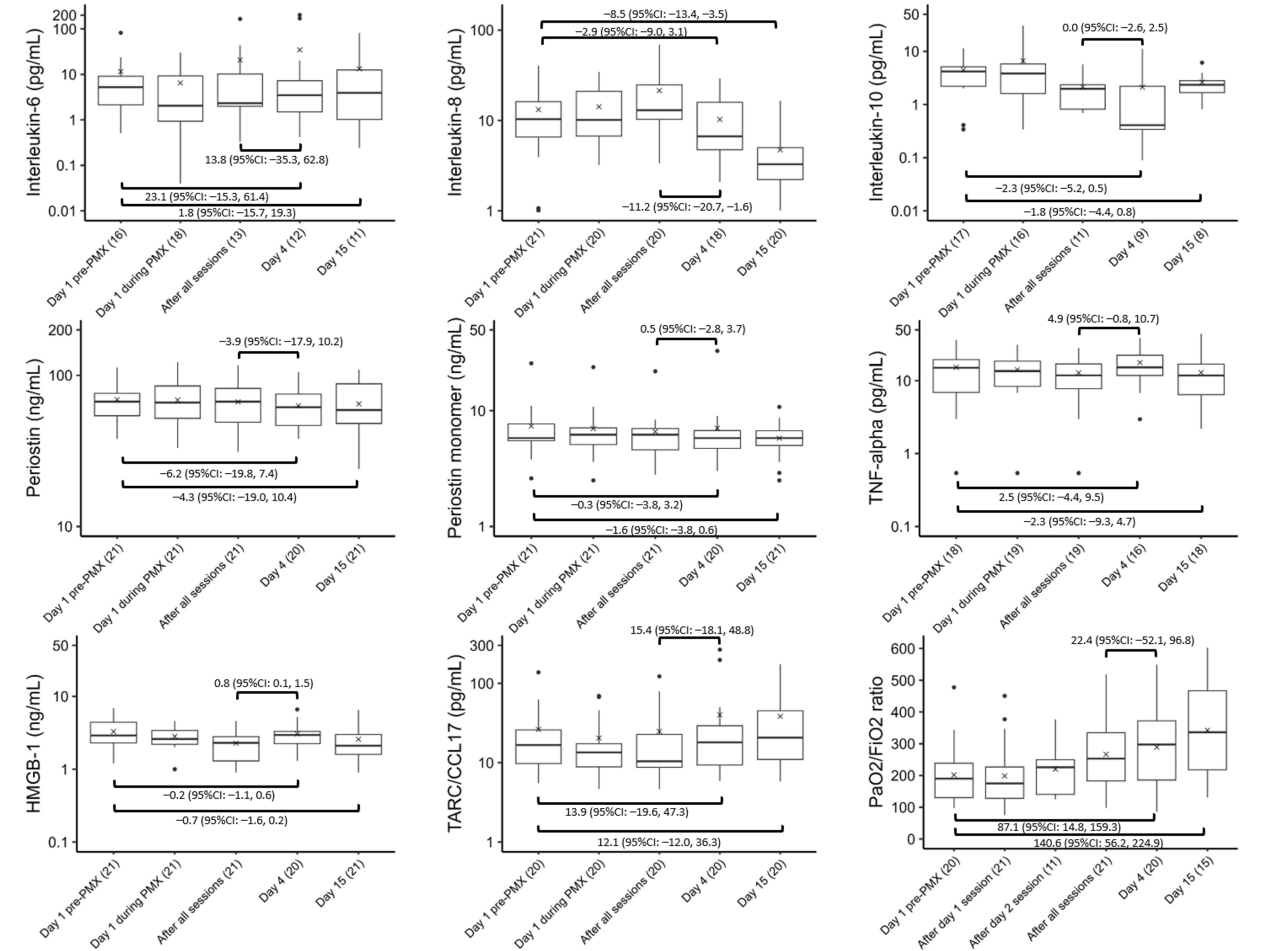
Trends in cytokines and the P/F ratio Variations in the levels of (A) IL-6, (B) IL-8, (C) IL-10, (D) periostin, (E) periostin monomer, (F) TNF-alpha, (G) HMGB-1, (H) TARC/CCL17, and (I) P/F ratio before, during, and after PMX treatment. The horizontal black bars indicate the first, second (median), and third quartiles at each time point, and the x marks indicate mean values. The values in each sub-figure are differences between means of two-time points, corresponding 95% confidence intervals. The numbers shown with () in the x-axis labels indicate the number of observations.

Figure 2 depicts the temporal changes in IL-6, IL-8, IL-10, periostin, periostin monomer, TNF-α, HMGB-1, TARC/CCL17, and the P/F ratio. With regard to improvements in oxygenation, the P/F ratio significantly improved on Days 4 (87.1, 95% CI: 14.8 to 159.3) and 15 (140.6, 95% CI: 56.2 to 224.9) relative to that at baseline.

We observed no significant changes in IL-6, periostin, periostin monomer, TNF-α, HMGB-1, and TARC/CCL17 between baseline and Day 4 or 15. However, the IL-8 level was significantly lower on Day 15 (−8.5, 95% CI: −13.4 to −3.5), while the IL-10 level was significantly lower on Day 4 (−2.3, 95% CI: −5.2 to 0.5) than at baseline. After all the PMX-DHP sessions and on Day 4, a significant decrease in the IL-8 level (−11.2, 95% CI: −20.7 to −1.6) was observed, whereas HMGB-1 levels showed a significant increase (0.8, 95% CI: 0.1 to 1.5).

Coagulation activation markers

**Figure 3:**
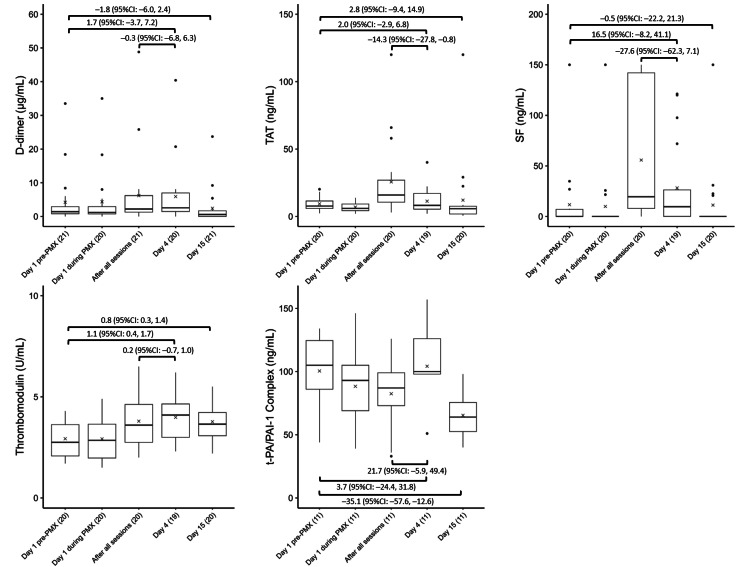
Trends in coagulation factors Variations in coagulation factors, including (A) D-dimer, (B) TAT, (C) SF, (D) thrombomodulin, and (E) t-PA-PAI-1 complex, before, during, and after PMX treatment. The horizontal black bars indicate the first, second (median), and third quartiles at each time point, and the x marks indicate mean values. The values in each sub-figure are differences between means of two-time points, corresponding 95% confidence intervals. The numbers shown with () in the x-axis labels indicate the number of observations.

Figure 3 shows the temporal changes in D-dimer, TAT, SF, thrombomodulin, and t-PA-PAI-1 complex levels. No significant D-dimer or SF changes were observed between baseline and Day 4 or 15. However, thrombomodulin significantly increased on Days 4 (1.1, 95% CI: 0.4 to 1.7) and 15 (0.8, 95% CI: 0.3 to 1.4). Furthermore, the TAT level significantly increased on Day 15 (2.8, 95% CI: −9.4 to 14.9). The t-PA-PAI-1 complex significantly decreased on Day 15 (−35.1, 95% CI: −57.6 to −12.6), while the TAT level significantly decreased after all sessions and on Day 4 (−14.3, 95% CI: −27.8 to −0.8).

Microbiota-derived peptides

**Figure 4:**
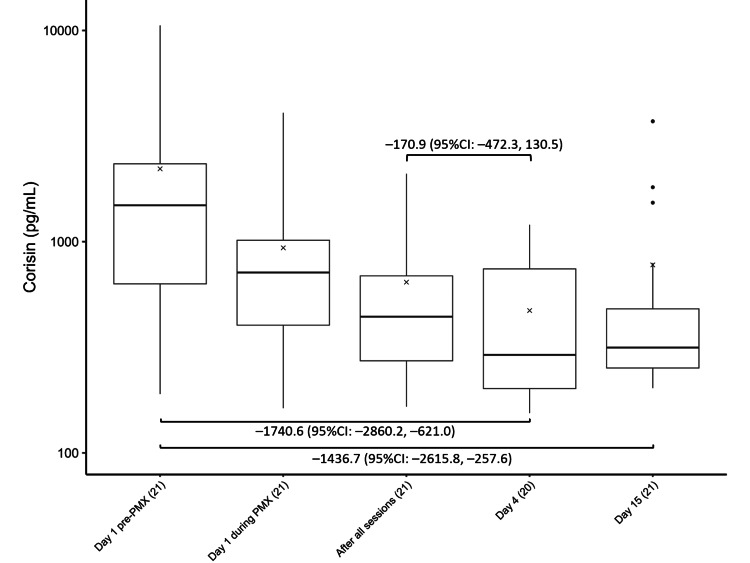
Trends in corisin levels Changes in corisin levels before, during, and after PMX treatment. The horizontal black bars indicate the first, second (median), and third quartiles at each time point, and the x marks indicate mean values. The values in each sub-figure are differences between means of two-time points, corresponding 95% confidence intervals. The numbers shown with () in the x-axis labels indicate the number of observations.

Figure 4 shows the time course of the corisin levels. Corisin levels were significantly lower on Days 4 (−1740.6, 95% CI: −2860.2 to −621.0) and 15 (−1436.7, 95% CI: −2615.8 to −257.6) than at baseline.

## Discussion

In this study, we evaluated the temporal changes in the P/F ratio, cytokines, coagulation markers, and a microbiota-derived proapoptotic peptide in COVID-19 patients requiring oxygenation and treated with PMX-DHP. IL-8 and IL-10 levels decreased after PMX-DHP, suggesting a transient modulation of inflammatory cytokines. Additionally, the levels of thrombomodulin and TAT increased, while T-PA-PAI-1 and microbiota-derived corisin levels decreased, suggesting that markers of the coagulation system and microbiota-derived peptides were also regulated. The significant improvements in the P/F ratio and reductions in IL-8 and corisin levels suggest that PMX-DHP may mitigate the inflammatory and apoptotic pathways associated with COVID-19 progression. These findings indicate a potential therapeutic role for PMX-DHP in improving oxygenation and preventing further respiratory deterioration.

Few previous reports have measured changes in biomarkers before and after PMX-DHP treatment in COVID-19 patients, and none have found a significant reduction in IL-6 levels [13, 16]. The present study showed no significant decrease in IL-6 levels, which is consistent with previously reported findings. A potential explanation for this observation is the ethnic background. Japanese COVID-19 patients have reduced levels of inflammatory markers (e.g., CRP) and cytokines (e.g., IL-6), as well as decreased activation of the coagulation and fibrinolysis systems compared with patients from other countries [17]. Moreover, the use of steroids may explain this finding. The present study included patients treated with steroids before the initiation of PMX-DHP treatment. Therefore, the previous treatment could have reduced the levels of some inflammatory cytokines, such as IL-6, IL-10, and TNF-α, before initiating PMX-DHP treatment. The median IL-6 concentration was less than 10 pg/mL, which may have prevented a clear indication of treatment-induced changes in levels. Another explanation for this finding is the inclusion of patients with mild COVID-19. In the present study, we included patients with a P/F ratio between 100 and 300, which might have resulted in the enrollment of patients with mild respiratory failure compared to the participants of a previous study [16]. Regarding changes in the levels of IL-8, the elevation of IL-8 in COVID-19 patients did not persist throughout the hospitalization period. The peak IL-8 level in patients with COVID-19 occurs during the active infection period when the viral load is high and decreases during recovery [18]. In the present study, IL-8 levels decreased on Day 15. This decrease was probably associated with improved patient condition due to reduced viral load. However, the results of the present study suggested that PMX-DHP had limited efficacy on cytokines.

SARS-CoV-2 infects cells by binding its spike protein to the angiotensin-converting enzyme 2 (ACE2). Furthermore, the presence of the virus has been demonstrated in endothelial cells that express ACE2 [19]. Viral infection of endothelial cells may lead to the disruption of the endothelial cell membrane. COVID-19 causes a nine-fold increase in microthrombus formation and intussusceptive angiogenesis in the lungs compared to influenza-induced pneumonia [20]. These pathological processes reduce ACE activity and cause an AngII/Ang1-7 imbalance, leading to vasculitis and thrombosis [21]. Viral infection-induced vascular endothelial injury is associated with increased production of PAI-1 by endothelial cells, which may explain the increased concentration of PAI-1 in COVID-19 patients [22]. Our study found that t-PA-PAI-1 levels in patients who underwent PMX-DHP increased on Day 4 but decreased on Day 15. This observation suggests that vascular endothelial cell damage caused by COVID-19 persists for a long time and that PMX-DHP treatment temporarily reduces t-PA-PAI-1 levels; however, these levels rebound after the discontinuation of PMX-DHP, as observed on Day 4. Increases in TAT and SF levels may reflect early coagulation activation. However, we observed a decreasing trend in these markers on Day 4 compared with post-PMX-DHP treatment, suggesting that PMX-DHP may improve the coagulation state. The transient decrease in t-PA-PAI-1 and increase in thrombomodulin levels may reflect a temporary stabilization of endothelial dysfunction. This suggests that PMX-DHP could play a role in modulating endothelial injury, a critical component of COVID-19-associated coagulopathy.

Large-scale genome-wide association studies revealed a genetic connection between IPF and severe COVID-19 [23]. The beneficial effects of PMX-DHP on lung oxygenation during acute exacerbations of interstitial lung diseases, including IPF, have been documented [11,24]. A study that measured cytokine changes after PMX-DHP treatment for acute exacerbation of IPF found no changes in IL-6 and IL-8, but significant decreases in IL-9, IL-10, IL-12, vascular endothelial growth factor (VEGF), and various other cytokines [25]. Recent research has suggested that corisin, a peptide secreted by various bacteria, including Staphylococcus spp, plays a role in the pathogenesis of acute exacerbations of IPF [15, 26]. These studies have shown that patients with acute exacerbations of IPF exhibit higher serum and bronchoalveolar lavage fluid (BALF) levels than those with stable disease. They have also proposed that corisin contributes to pulmonary fibrosis progression by inducing excessive inflammation mediated by MCP-1 and TNF-α and triggering apoptosis in alveolar epithelial cells. Our present study showed that TNF-α levels did not change before and after PMX-DHP treatment. However, the corisin levels declined early. This finding suggests that the therapeutic efficacy of PMX-DHP may involve various mediators in addition to inflammatory cytokines such as TNF-α. This modulation may prevent alveolar epithelial cell apoptosis by reducing corisin levels.

This study demonstrates that PMX-DHP treatment in oxygen-dependent COVID-19 patients leads to significant biomarker changes, aligning with its proposed mechanism of action in modulating inflammation, coagulation, and microbiota-derived mediators. These results can be interpreted as supporting the hypothesis that PMX-DHP may modulate key pathophysiological pathways in severe COVID-19.

This study had several limitations. First, it lacked a control group that did not receive PMX-DHP treatment. This prospective study was conducted when the pandemic was rapidly spreading, and no effective treatments or vaccines were available. Therefore, we used a single-arm design to avoid reducing the number of patients who could benefit from PMX-DHP treatment. Second, we cannot rule out the possibility that changes in various mediators were influenced by other medications, such as steroids or heparin, rather than PMX-DHP. While the observed changes in biomarkers suggest a therapeutic effect of PMX-DHP, the single-arm design and use of concomitant medications like corticosteroids limit the ability to definitively attribute these effects to the intervention. However, it is important to note that many patients in this study had already received steroids or immunomodulatory drugs before starting the PMX-DHP treatment. Therefore, the demonstration of significant changes in inflammation, coagulation, and microbial mediators after treatment with PMX-DHP is an important contribution to the research field.

One of the strengths of our study is that it is among the first to investigate the effects of PMX-DHP on a comprehensive range of inflammatory, coagulation, and microbiota-derived markers in oxygen-dependent COVID-19 patients. The longitudinal design enables the assessment of temporal trends, while the multicenter approach improves the generalizability of the findings. Additionally, the inclusion of biomarkers such as corisin, which have been rarely studied in the context of COVID-19, offers novel insights into the disease’s pathophysiology and potential therapeutic mechanisms. On the other hand, a limitation of our study is its single-arm design, which was necessitated by the urgency of the pandemic. This design restricts the ability to establish causal inferences regarding the efficacy of PMX-DHP. Nevertheless, the significant biomarker changes observed post-treatment suggest its potential therapeutic role. To validate these findings and address the potential confounding effects of concomitant medications, future randomized controlled trials are essential.

## Conclusions

The present study demonstrated changes in the circulating levels of cytokines, coagulation activation markers, and microbiota-derived proapoptotic corisin in patients with COVID-19 who required supplemental oxygen and were treated with PMX-DHP. Although the therapeutic effects of PMX-DHP on inflammatory cytokines may be limited, our findings suggest that PMX-DHP may have therapeutic benefits by reducing the activation of the coagulation system and proapoptotic peptides from the microbiota. This is a significant finding, as coagulopathy and dysbiosis are major complications of COVID-19. These findings provide preliminary evidence for the role of PMX-DHP in improving oxygenation and modulating key inflammatory and coagulation pathways in COVID-19. Future randomized controlled trials are needed to confirm these effects and evaluate their impact on clinical outcomes such as mortality and recovery rates.
